# Draft Genome Sequence of the Nonmarine Agarolytic Bacterium *Cellvibrio* sp. OA-2007

**DOI:** 10.1128/genomeA.00468-15

**Published:** 2015-05-14

**Authors:** Syazni, Mitsunori Yanagisawa, Masahiro Kasuu, Kiyohiko Nakasaki, Osamu Ariga

**Affiliations:** a Department of International Development Engineering, Graduate School of Engineering, Tokyo Institute of Technology, Tokyo, Japan; b Department of Environmental Sciences, School of Food, Agricultural and Environmental Sciences, Miyagi University, Miyagi, Japan; c School of Environmental Science and Engineering, Kochi University of Technology, Kochi, Japan

## Abstract

*Cellvibrio* sp. OA-2007 is a Gram-negative, aerobic, and agarolytic bacterium isolated from activated sludge. We present the draft genome sequence of strain OA-2007, composed of 97 contigs, totaling 4,595,379 bp in size, and containing 4,094 open reading frames, with a G+C content of 47.71%.

## GENOME ANNOUNCEMENT

Agarases are important enzymes in the agar metabolic pathway, with various applications. They have been used to hydrolyze agar into oligosaccharides and monosaccharides such as galactose and 3,6-anhydro-l-galactose. The oligosaccharides produced by agarose hydrolysis have been reported to exhibit antioxidant activity (1) and to have moisturizing and whitening effects on skin (2). Galactose produced from agarose has been used for bioethanol production (3), and 3,6-anhydro-l-galactose has been reported to have skin-whitening and anti-inflammatory activities (4). Agarases also have other experimental uses, such as isolation of protoplasts from seaweeds (5) and recovery of DNA fragments from agarose gels (6). Thus, since agarases have many applications, many studies have been published on various agarolytic bacteria, their agarase enzymes, and the genes that encode them (7).

An agarolytic bacterium, *Cellvibrio* sp. OA-2007, was isolated from activated sludge, and the gene encoding β-agarase, an agarase that hydrolyzes β-glycosidic bonds in agarose and produces neoagaro-oligosaccharides, was successfully cloned (8). In addition, an α-neoagaro-oligosaccharide hydrolase enzyme, which hydrolyzes neoagaro-oligosaccharides, was purified and characterized (9). To obtain data on other agarase enzymes and the genes that encode them, we analyzed the genome sequence of strain OA-2007.

Genomic DNA was extracted from strain OA-2007, and the genome was sequenced by Macrogen Japan (Tokyo, Japan), using an Illumina HiSeq 2000 system. A total of 20,400,678 reads (2,060,468,478 bases) were obtained and assembled using the SOAPdenovo2 version 2.04-r240 assembly software (10). The assembled draft genome is 4,595,379 bp in size and contains 97 contigs with an *N*_50_ length of 225,384 bp. The average, longest, and shortest contigs are 47,375 bp, 404,899 bp, and 141 bp, respectively. The G+C content of this assembled draft genome is 47.71%. Moreover, 4,094 open reading frames were identified using the Glimmer system (11) and annotated using BLAST.

Five putative agarase genes were detected in the genome sequence of strain OA-2007. Furthermore, 27, 12, 9, and 4 genes corresponding to enzymes for degradation of other polysaccharides (glucan, xylan, pectate, and alginate, respectively) were also identified. Of the translation products of these putative genes, 10 glucan-degrading enzymes, 5 xylan-degrading enzymes, and 1 pectate-degrading enzyme showed high homology to the corresponding proteins derived from *Cellvibrio* sp. BR. These results indicate that strain OA-2007 has the ability to degrade not only agar but also other polysaccharides and could be useful for degradation of the polysaccharides in biomass. Recombinant microorganisms carrying genes encoding polysaccharide-degrading enzymes from strain OA-2007 may be useful for the production of many chemicals from biomass materials in biorefineries.

### Nucleotide sequence accession numbers. 

The draft genome sequence of strain OA-2007 was submitted to DDBJ under the accession numbers BBUL01000001 to BBUL01000097.
